# Lead Excretion in Spanish Children with Autism Spectrum Disorder

**DOI:** 10.3390/brainsci5010058

**Published:** 2015-02-16

**Authors:** Milagros Fuentes-Albero, Carmen Puig-Alcaraz, Omar Cauli

**Affiliations:** 1 Children’s Mental Health Centre, Hospital Arnau de Villanova, 46015 Valencia, Spain; E-Mail: milafuentesalbero@yahoo.es; 2 Area of Mental Health and Psychiatry, Area Sagunto, 46520 Sagunto (Valencia), Spain; E-Mail: pppajaron@yahoo.es; 3 Department of Nursing, University of Valencia, 46010 Valencia, Spain

**Keywords:** lead, autism spectrum disorder, core symptoms, biomarker, DMS IV

## Abstract

Among epigenetic factors leading to increased prevalence of juvenile neuropsychiatric disorders, including autism spectrum disorder, exposure to metals, such as lead (Pb) have led to conflicting results. The aim of the present study was to determine the levels of Pb in the urine of children with autism spectrum disorder (ASD) compared with typically developing children (TD) age- and sex-matched, and to analyze any association between core symptoms of ASD, special diets, supplements intake or prescription drugs and the concentration of Pb. The study was performed in a group of children with ASD (*n* = 35, average age 7.4 ± 0.5 years) and TD (*n* = 34, average age 7.7 ± 0.9 years). Measurement of lead in urine was performed by atomic absorption spectrometry; symptoms of ASD were analyzed by diagnostic and statistical manual of mental disorders (DMS-IV) using the questionnary ADI-R. Careful clinical evaluation was also undertaken and statistical analysis was done taking into account any possible confounding factor.

## 1. Introduction

Autism spectrum disorder (ASD) is a severe developmental disorder in the majority of cases, which involves social withdrawal, communication deficits, and stereotypic/repetitive behavior. The pathophysiological etiology that precipitates autism symptoms remains elusive and controversial in many cases, but both genetic and environmental factors (and their interactions) have been implicated [1,2]. One environmental factor that has received significant attention is exposure to lead (Pb) [2,3,4], a known neurotoxic agent with no “safe” threshold levels in children.

Pb is a non-essential toxic heavy metal widely distributed in the environment and chronic exposure to low levels of Pb has been a matter of public health concern in many countries. Despite improvements in public health policies and substantial reductions in blood Pb levels, Pb exposure remains an important health problem worldwide. Accumulating evidence suggests a link between Pb exposure and memory impairment [3]. Moreover, it has been reported that the degree of performance impairment over time increased with increasing bone Pb concentration, a marker of cumulative exposure [4]. Additionally, increased Pb concentration in bone is associated with reduced scores on the Mini Mental Status Exam [5,6]. A study of elderly women reported inverse associations between reaction time, digit symbol and trail making, with blood Pb levels, and another longitudinal community study reported an inverse relationship between cumulative Pb exposure and performance in eight cognitive tests [7,8]. Pb may be found in the dirt near roads and can still be found in paint from older houses but it varies depending on geographical locations or socio-economical status [9,10]. In Spain, diet and tap water are currently considered the major contributing factor to Pb exposure [11,12]. Other sources contributing to Pb exposure are the changes in water treatment (switching from chlorine to chloramine use, to avoid the production of disinfection by-products) may increase the amount of dissolved lead in water [13]. Although exposure to other Pb sources could be more relevant, sources from Pb-based paint in old houses has also been reported in Spain [14]. The decreased concentrations in air and in children in the last decade has been most likely the result of legislative measures regulating the maximum amount of Pb in gasoline in 1987 until a complete ban in August 2001 [15]. Elevated Pb levels have been reported in biological fluids (blood and urine) and hair in several juvenile onset neuropsychiatric disorders such as ASD and attention-deficit hyperactivity disorder (ADHD). However, these investigations led to mixed results ranging from increases to no changes [16,17,18,19] probably because they have been performed in populations differentially exposed to Pb. In addition, some children with ASD can eat paint chips or display pica behavior leading to an increased exposure to Pb.

Therefore, in this study we assessed three main objectives:

(1) To measured urinary Pb concentration in children with ASD and typically developing (TD) children.
(2) Next, we evaluated the relationship between urine Pb levels and the severity of core symptoms of ASD (deficit in communication, socialization and restricted/stereotyped activities) according to the fourth edition of the Diagnostic and Statistical Manual of Mental Disorders (DMS-IV).
(3) Finally, we studied the influence of several factors such as age, sex, medication, diet, or other neurological comorbidities (sleep or gastrointestinal problems, epilepsy, food allergies/intolerances, level of verbality, *etc.*) in these associations in order to find any relationship with urinary Pb concentration.

## 2. Experimental Section

### 2.1. Participants

Study participants were recruited from patients attending specialized and qualified centers for psychotherapeutic intervention in children with ASD. All these centers are located in Valencia (Spain); data were collected between September 2012 and November 2013. All children had previously been diagnosed with ASD (including autistic disorder and pervasive developmental disorder not otherwise specified) by a clinical psychologist/psychiatrist. ASD was confirmed using the DMS-IV diagnostic criteria using a standard neurodevelopment examination and interview. In addition, all other relatively common genetic or neurological conditions responsible for syndromic ASD were ruled out. Parents’ consent was required before DMS-IV interview, medical evaluation and to anonymously extract their child’s medical history, physical examination findings, and neurological and metabolic test results into a clinical database.

The study was conducted in accordance with the Declaration of Helsinki, and the protocol was approved by the Ethics Committee of University of Valencia (H1397475950160).

### 2.2. Evaluation of Core Symptoms

All children were previously diagnosed with an ASD using the criteria for the ICD-10 (tenth revision of the international statistical classification of diseases and related health problems: *i.e.*, the DSM-IV). Core symptoms of autism based on the DMS-IV were evaluated with the revised autism diagnostic interview™ (ADI-R). The first section of the interview is used to assess the quality of social interaction and includes questions about emotional sharing, offering and seeking comfort, social smiling, and responding to other children. The communication and language behavioral section investigates stereotyped utterances, pronoun reversal, and social usage of language. Stereotyped utterances are the few words or sounds that the individual uses and repeats most often. The restricted and repetitive behaviors section includes questions about unusual preoccupations, hand and finger mannerisms, and unusual sensory interests. Cut off scores are 10 for social interaction; 8 for communication and language, if verbal, and 7 if non-verbal; and 3 for restricted and repetitive behaviors. This diagnostic tool has a high correlation level with the diagnostic criteria reported in the DSM-IV and CIE-10 and has good psychometric properties (96% sensitivity and 92% specificity) [20,21].

### 2.3. Medical Interview and Evaluation

Extensive medical histories of the autistic and control children were taken, *i.e.*, detailed history of pregnancy and labor, morphometric measures at birth, Apgar scores, vaccinations, mental and motor development, any illnesses or traumatic events, diet, body mass index, head circumference, vitamin or supplement intake, medication, epilepsy, gastrointestinal or sleep problems, regression, food allergies or intolerances, and laboratory results about syndromic causes of ASD (fragile X, chromosomopathy, genetic diseases, *etc.*).

### 2.4. Pb Analysis

On the first morning urine samples were collected, from 35 autistic children (age 4–13 years) and from 34 neurologically healthy children (age 4–12 years). The samples were centrifuged at 13,000 rpm for 10 min at 4 °C and the resulting supernatants were immediately stored at −80 °C until analysis. To prepare the sample for Pb measurement, a 0.2-mL aliquot of urine was mixed well with 3.9 mL of 0.5 N nitric acid containing 0.01% Triton X-100. After centrifugation the supernatant was taken. To determine Pb concentration in samples, the same volume of each samples and 0.2% magnesium nitrate (as a modifier) was mixed and 10 μL was injected into graphite furnace of atomic absorption spectrophotometer (Perkin-Elmer 3030) [22]. Concentrations of Pb were also analyzed as a normalized ratio to urinary creatinine concentration or to urine density in order to account for glomerular filtration rate changes.

### 2.5. Statistical Analysis

The results are presented as the mean ± the standard error mean (SEM). Shapiro-Wilk test was used to check data distribution and the unpaired *t*-test to compare mean values for Pb in the ASD and TD groups. Correlation analysis was performed with the non-parametric Spearman test and correlation between any of the confounding factors was also analyzed using logistic regression bivariate analysis. The level of statistical significance was set at 95% confidence (*p* < 0.05). Statistical analysis was performed using SPSS software (version 22.0; SPSS, Inc., Chicago, IL, USA).

## 3. Results

### 3.1. Description of the Sample, Evaluation of Core Symptoms and Other Clinical Features

In the ASD group, there were 25 boys (71%) and 10 girls (29%), ranging in age from 4 to 13 years (mean: 7.4 ± 0.5 years). The control group for the baseline metabolite concentrations consisted of 34 apparently healthy age-matched children who had no previous history of developmental delay or neurological symptoms (mean age 7.7 ± 0.9 years). Mean body mass index was 18.4 ± 0.5 and the mean head circumference percentile rank was 61 ± 5 cm. Thirty patients had autistic disorder and five patients had pervasive developmental disorder not otherwise specified. ADI-R gave a mean score of 20.9 ± 2.1 for socialization deficits; 22.4 ± 2.9 for communication deficits, and 7.2 ± 1.1 for repetitive/restricted behaviors. The total ADI-R score was 50.4 ± 4.4 and the score range was 17–80. Almost 57% children had sleep problems (Table 1). Gastrointestinal disorders were the most common associated medical condition; 77% had past or current gastrointestinal problems, the most common complaint being constipation followed by diarrhea. Gastric reflux and cycling vomiting were reported each in one case. Regression occurs when a child appears to develop normally but then starts to lose speech and social skills, typically between the ages of 15 and 30 months, and is subsequently diagnosed with ASD. Regressive autism was referred in 43% cases, food allergy/intolerance in 31%, incidences during pregnancy or labor in 24% cases, low Apgar scores (less than 8) in 11%, minor morphological abnormalities (dysmorphic traits such as asymmetric eyes, low-set ears, a prominent forehead, flat philtrum, thin upper lip, fifth finger clinodactyly, brachycephaly, or open mouth appearance) in 34% of the sample and recurrent infection during first year of life was reported in 10 children. The antipsychotic drug risperidone was the drug mostly prescribed (23% of sample), followed by methylphenidate and atomoxetine (8% and 11% of sample, respectively). The antiepileptic drugs (valproic acid, lamotrigine, or levetiracetam) were prescribed in 10% of the sample, and melatonin was prescribed or taken electively at the volition of parents in 26% of the sample. Administration of supplements containing vitamins, minerals, omega-3/6 acids, or folic acid ranged between 11% and 22% of the sample. Thirty-one percent of the sample was on a special diet, namely casein-free and/or gluten-free.

**Table 1:** Demographic, morphometric and clinical features of the study population.

**Age (years)**	ASD children: 7.4 ± 0.5
	range (4–13 years)
	TD children: 7.7 ± 0.9
	Range (4–13 years)
**Gender**	ASD children: 25 boys 10 girls
	TD children: 24 boys 10 girls
**Body mass index**	ASD children: 18.4 ± 0.5
	range (14–25)
	TD children 18.7 ± 0.8
	(range 15–26)
**Head circumference (percentile rank)**	61 ± 5
	range (2–100)
**ASD type**	30 Autism, 5 NOS-PPD
**Score of socialization deficit (ADI-R subscale)**	20.9 ± 2.1
**Score of communication deficit (ADI-R subscale)**	22.4 ± 2.9
**Score of repetitive/restricted behaviours (ADI-R subscale)**	7.2 ± 1.1
**Total ADI-R score**	50.4 ± 4.4

### 3.2. Evaluation of Urinary Pb Concentration

**Figure 1:**
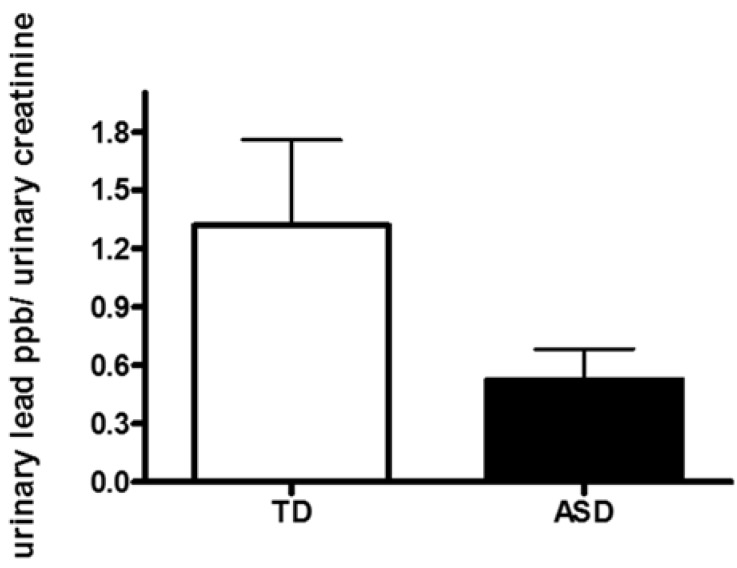
Urinary concentration of lead (Pb) in children with autism spectrum disorder (ASD) and typically developing children (TD).

No statistically difference was observed between urinary concentration of Pb in children with ASD compared to TD children (0.60 ± 0.19 *versus* 1.32 ± 0.44 ppb/mg·dL^−1^, respectively *p* = 0.09; Figure 1). In the ASD group there was a children with 5.59 ppb/mg·dL^−1^ and in the TD group two children with 12.76 ppb/mg·dL^−1^ and 6.41 ppb/mg·dL^−1^ (Figure 1). Even analyzing data without with these high values the comparison between the two groups is still no significant (*p* = 0.08) with a tendency to higher urine Pb level in TD group. By normalizing urinary Pb concentration to urinary specific gravity or to urine creatinine concentration we obtained similar results (no significant change in urinary Pb concentration in ASD compared to TD children) (data not shown). Sex-specific analyses for the three urinary parameters showed no significant differences suggesting that the results are not due to spurious sex-specific differences. None of the children had pica behavior or ferropenic anemia that could have caused an increase in Pb exposure or absorption, respectively.

### 3.3. Correlation between Pb and Core Autism Spectrum Disorder Symptoms

**Figure 2:**
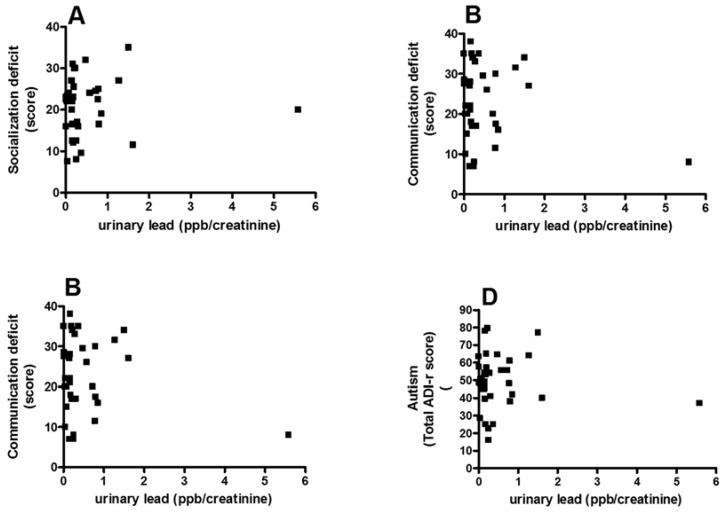
Correlation between the score in the socialization (**A**); communication (**B**); and stereotyped/restricted activities (**C**) and total the revised autism diagnostic interview™ (ADI-R) autism score (**D**) and urinary concentration of Pb.

No significant correlation was found between socialization deficit scores assessed with ADI-R and the concentration of Pb in urine (Spearman *r* = 0.12, *p* = 0.54; Figure 2A) or with the communication deficit scores in urine (Spearman *r* = 0.08, *p* = 0.67; Figure 2B) or with repetitive/restricted behavior scores (Spearman *r* = 0.21, *p* = 0.27; Figure 2C). No significant correlation was found between total ADI-R score and Pb concentration (Spearman *r* = 0.06, *p* = 0.76; Figure 2D) with the concentration of Pb in urine in children with ASD.

### 3.4. Analysis of Possible Confounding Factors

The lack of correlation between Pb concentration and autism score was maintained after controlling for several factors: age (Spearman *r* = 0.15, *p* = 0.11); sex (Spearman *r* = 0.04, *p* = 0.13); past or current intestinal problems (mainly constipation or diarrhea; Spearman *r* = −0.05, *p* = 0.91); food allergy or intolerance (Spearman *r* = 0.03, *p* = 0.85); sleep problems (mainly difficulty in starting to sleep or early nocturnal awakening; Spearman *r* = 0.14, *p* = 0.25); vitamin supplement (Spearman *r* = −0.06, *p* = 0.19); DHA (omega 3 fatty acid) supplementation (Spearman *r* = −0.03, *p* = 0.55); mineral supplement (Spearman *r* = −0.02, *p* = 0.16); regression (Spearman *r* = 0.02, *p* = 0.31); psychotropic drugs (Spearman *r* = 0.17, *p* < 0.16), and the presence of verbal language (Spearman *r* = 0.02, *p* = 0.18). Multivariate analysis gave similar results.

## 4. Discussion

The particular vulnerability of the developing nervous system to low-level exposure to chemicals is well established. Developmental neurotoxicity has been reported for a large number of industrial chemicals and metals, and in particular the case of Pb there is not a safe threshold level and its concentration should be actually zero in biological fluids and tissues. The concentration of Pb in both ASD and TD children are within the reference range reported in the literature for unexposed general population [23,24,25,26,27]. In order to set clinical vigilance and monitor toxicants levels that can contribute or to worsen symptomatology in children with ASD is pivotal to know whether increased levels of Pb occur in children with ASD. Pb levels have been declining steadily in the environment since the last decades when Pb additives to gasoline were phased out, whereas the incidence of ASD has been rising in the last decade [28]. Thus, it has been assumed that exposure to Pb likely does not account for the increasing incidence of ASD observed in the last decade, as the last studies reporting an incidence of ASD around 1% and is on the rise [29]. However it cannot be ruled out that ongoing Pb exposure can worsen symptomatology in children with ASD and this effect needed to be tested. In addition, the most significant purpose of our study was to assess whether there is a relationship between lead concentration and the core symptoms of ASD. Our result clearly show that an increased lead concentration is not accompanied by an increased symptomatology in any of the three core symptoms of ASD according to DMS IV criteria [30].

Regarding the question whether an increased lead burden is found in children with ASD compared to TD group, our study agrees with other studies that find no differences of lead concentrations in biological fluids or tissues. No significant differences were found in blood Pb levels between in children ASD and TD aged three to four years in a study performed in U.S. [18]. The lack of an increased lead concentration in blood in children with ASD has been demonstrated in a study performed in Slovenia [31]. Pb levels in baby teeth and scalp hair of children with autism spectrum disorder and typically developing children led to similar results [26,32,33]. However, another study performed in U.S. found a significant increase of lead concentration in children with ASD compared to TD in urine and red blood cells [33]. One study performed in Turkey also showed an increased urinary excretion in children with ASD [18]. The discrepancy between the results obtained in our study and those reporting an increase excretion of lead in children with ASD may be due to differences in geographic exposure to Pb (Spain *vs.* Arizona) or rather to the socio-demographic variables or clinical features of the study sample (living in old houses with Pb-based paint or located close to industrialized areas, low income social class, children with pica behavior, ferropenic anemia, *etc.*). However, the age and gender of the controls were unspecified in the study performed in Turkey and may not have matched those of the ASD group or other factors mentioned above might have played a role.

We also analyzed whether there was any factor that could mediate an eventual correlation between Pb and core symptoms of ASD. To this aim, we performed a detailed statistical analysis in order to evaluate if correction for sociodemographic, morphometric-features, type of comorbidities, prescribed drugs or intake or supplements supplements can give rise to significant correlation between Pb concentration and individual core symptom or total score of ASD. The lack of significant association between Pb and these confounding factors supports that an increased Pb concentration does not play a role in ASD symptomatology in Spain.

However, the limited size of our sample means that further analysis will be required to clarify this finding and we cannot generalize the results for all children with ASD until longitudinal studies will be performed in order to establish any possible causal relationship. We cannot rule out that the parents of children with ASD may have cleaned up the diet and environment so that there was less lead exposure. Another limitation is that our study took place in our country and the conclusions might not apply to other countries. Though it seems unlikely that the findings of this study directly relate exposure to Pb to the symptoms of ASD, however it is unknown whether Pb can have differential effects on cell function, different gene transcription cells in ASD patients [18].

## 5. Conclusions

Our results showed the excretion of Pb is unaffected in children with autism spectrum disorder (ASD). The concentration of Pb in urine does not correlate with any core symptoms in children with ASD.
